# Episodic memory performance in a multi-ethnic longitudinal study of 13,037 elderly

**DOI:** 10.1371/journal.pone.0206803

**Published:** 2018-11-21

**Authors:** Seonjoo Lee, Xingtao Zhou, Yizhe Gao, Badri Vardarajan, Dolly Reyes-Dumeyer, Kumar B. Rajan, Robert S. Wilson, Denis A. Evans, Lilah M. Besser, Walter A. Kukull, David A. Bennett, Adam M. Brickman, Nicole Schupf, Richard Mayeux, Sandra Barral

**Affiliations:** 1 Research Foundation for Mental Hygiene and the Department of Biostatics, College of Physicians and Surgeons, Columbia University, New York City, New York, United States of America; 2 The Georgetown University Lombardi Comprehensive Cancer Center, Georgetown University, Washington, D.C., United States of America; 3 The Department of Neurology, College of Physicians and Surgeons, Columbia University, New York City, New York, United States of America; 4 The Taub Institute for Research on Alzheimer's Disease and the Aging Brain, Columbia University College of Physicians and Surgeons, New York City, New York, United States of America; 5 Gertrude H. Sergievsky Center and Department of Neurology, Columbia University College of Physicians and Surgeons, New York City, New York, United States of America; 6 Department of Public Health Sciences, University of California at Davis, Davis, California, United States of America; 7 Rush Alzheimer’s Disease Center, Rush University Medical Center, Chicago, Illinois, United States of America; 8 Department of Behavioral Sciences, Rush University Medical Center, Chicago, Illinois, United States of America; 9 Department of Internal Medicine, Rush Institute for Healthy Aging, Rush University Medical Center, Chicago, Illinois, United States of America; 10 School of Urban and Regional Planning, Florida Atlantic University, Boca Raton, Florida, United States of America; 11 Department of Epidemiology, National Alzheimer's Coordinating Center, University of Washington, Seattle, Washington, United States of America; 12 Department of Epidemiology, Mailman School of Public Health, Columbia University, New York City, New York, United States of America; Nathan S Kline Institute, UNITED STATES

## Abstract

Age-related changes in memory are not uniform, even in the absence of dementia. Characterization of non-disease associated cognitive changes is crucial to gain a more complete understanding of brain aging. Episodic memory was investigated in 13,037 ethnically diverse elderly (ages 72 to 85 years) with two to 15 years of follow-up, and with known dementia status, age, sex, education, and *APOE* genotypes. Adjusted trajectories of episodic memory performance over time were estimated using Latent Class Mixed Models. Analysis was conducted using two samples at baseline evaluation: i) non-cognitively impaired individuals, and ii) all individuals regardless of dementia status. We calculated the age-specific annual incidence rates of dementia in the non-demented elderly (n = 10,220). Two major episodic memory trajectories were estimated: 1) Stable—consisting of individuals exhibiting a constant or improved memory function, and 2) Decliner—consisting of individuals whose memory function declined. The majority of the study participants maintain their memory performance over time. Compared to those with Stable trajectory, individuals characterized as Decliners were more likely to have non-white ethnic background, fewer years of education, a higher frequency of ε4 allele at *APOE* gene and five times more likely to develop dementia. The steepest decline in episodic memory was observed in Caribbean-Hispanics compared to non-Hispanic whites (p = 4.3 x 10^−15^). The highest incident rates of dementia were observed in the oldest age group, among those of Caribbean-Hispanics ancestry and among Decliners who exhibited rates five times higher than those with Stable trajectories (11 per 100 person-years versus 3 per 100 person-years. Age, education, ethnic background and *APOE* genotype influence the maintenance of episodic memory. Declining memory is one of the strongest predictors of incident dementia.

## Introduction

Aging can be associated with changes in memory function, even in the absence of mild cognitive impairment or dementia. Elderly cohort studies have shown that cognitive change with aging is very heterogeneous, with some individuals showing decline, and others remaining relatively stable over time. The nature and extent of age-related changes in memory function in older adults have been primarily studied in samples of individuals with neurological diseases [1–7], and fewer studies have focused in the dynamics of memory performance over time in cognitive healthy elderly [8–12]. Establishing the patterns of cognitive change among the elderly is crucial to gain a complete understanding of the aging brain, and may shed light on abnormal brain processes.

In one of the largest studies to date [13], different factors (age, sex, education, ethnic background, and the ε4 allele at the Apolipoprotein E (*APOE)* gene) potentially influenced decline across different cognitive domains (memory, processing speed, language, executive functioning, and the Mini-Mental State Examination), in a group of 42,170 middle and older adults from twelve different countries. Although there was considerable heterogeneity in the rates of cognitive decline across the cohorts, the direction of the associations between risk factors and cognitive decline appeared to be consistent across cohorts.

Despite the impressive sample size, the study authors mainly focused on overall trends of age-related cognitive decline, rather than on inter-individual variability of trajectories of cognitive performance.

Although there are several methodological approaches used in developmental cognitive neuroscience, growth curve models (GCM) represent a powerful analytical framework to model individual differences in cognitive change over time, as well as the variability of patterns of cognitive change between individuals [14]. In cognitive neuroscience GCMs have been derived using linear mixed effects model (LMEM) or latent curve models (LCM) [1–4, 6–11, 15–23]. LCM uses factor analysis and structural equation models for unobserved outcomes [14, 24] and are best suited for complex models with straightforward large data structures [25]. The flexibility of the LCM approach in incorporating variables with high degree of inter-individual variability (i.e. the number of follow-up visits), becomes especially useful to study trajectories of cognitive functioning in elderly cohorts in which individuals were enrolled at different ages and followed with different time intervals [26].

Longitudinal studies of cognitive function using LCM frameworks have consistently distinguished between those whose memory performance declines over time and those with a stable memory trajectory [3, 10–12]. Similar patterns have also been reported in persons with mild cognitive impairment (MCI) [6] and in persons with a diagnosis of Alzheimer’s disease (AD) [1].

The majority of previous studies using trajectories of cognitive performance have investigated non-Hispanic white (NHW) populations [1–4, 6–11, 15, 18, 19, 22, 23] and have relied on the Mini-Mental Examination (MMSE) as the measure of cognitive performance [17, 20, 21]. Although MMSE is one of the most widely used cognitive screening tests in clinical and epidemiological research, it is limited by sensitivity to practice effects, large ceiling and floor effects [27, 28] and insufficient assessment of specific cognitive domains. The MMSE uses a simple 3-item recall task as a measure of memory, which is less sensitive than targeting key cognitive domains such as episodic memory. Moreover, the most pronounced and consistent cognitive deficits in preclinical Alzheimer's disease are seen for tasks assessing episodic memory [29, 30]. However, estimation of cognitive trajectories using memory as an outcome measure in non-White or admixed populations has been limited [16, 17, 20, 21], and the largest study to date consists of 1,336 Mexican American adults 75 years of age and older [21].

The primary goal of our study was to identify trajectories for episodic memory performance in a large and ethnically diverse sample of older adults stratified by their dementia status at their first clinical evaluation (non-cognitively impaired participants and all participants);. The secondary goals of our study were: 1) to investigate whether socio-demographic factors (sex, ethnicity and education) and *APOE* genotype are predictors of age-related memory decline and 2) to investigate whether incident rates of dementia may differ when sample is stratified by age, sex, ethnicity and EMT clusters (EMT_Stables_ and EMT_Decliners_).

## Material and methods

### Study cohorts

Longitudinal data on episodic memory performance was gathered from five different study cohorts: The Washington Heights-Inwood Columbia Aging Project (WHICAP), The Chicago Health and Aging Project (CHAP), The National Institute on Aging Late-Onset Alzheimer Disease Family Based Study (NIA-LOAD), The National Alzheimer's Coordinating Center (NACC) and The Rush Alzheimer’s Disease Center cohorts (ROSMAP). Detailed descriptions of cognitive assessment within each of the study cohorts are presented below. The criteria to determine the clinical diagnosis of the dementia status of the study participants was the same across all studies and within study across all the participants’ evaluations. The diagnosis of Alzheimer's Disease is based on the National Institute of Neurological and Communicative Disorders and Stroke and the Alzheimer’s Disease and Related Disorders Association (NINCDS-ADRDA) criteria [31, 32]. Based on this clinical diagnosis of dementia status study participants were classified as with a diagnosis of AD or as non-cognitively impaired if they were found to have no evidence of dementia diagnosis.

#### The Washington Heights-Inwood Columbia Aging Project (WHICAP)

Participants were drawn from a multiethnic, population-based, prospective study of Medicare beneficiaries aged 65 and older residing in northern Manhattan [33]. All WHICAP participants provided written informed consent and the study procedures were approved by the Institutional Review Boards at Columbia University.

Assessment of Cognitive Function. Individual cognitive tests were grouped into cognitive domains based on previous factor analysis of the WHICAP neuropsychological battery [34]. The episodic memory domain was quantified as composite scores of standardized measures of total immediate recall, delayed recall, and delayed recognition trials from the Selective Reminding Test [35]. Raw scores were standardized using the sample’s means and standard deviations from entire WHICAP sample at baseline. Standardized scores were then averaged into the episodic memory domain.

#### The Chicago Health and Aging Project (CHAP)

The study sample consisted of participants from a longitudinal population-based study of persons aged 65 years and older, in a biracial neighborhood of Chicago. Written informed consent was obtained and the study was approved by the Institutional Review Board at Rush University Medical Center. Detailed description of the cohort can be found elsewhere [36].

Assessment of Cognitive Function. As previously described [37], scores of immediate and delayed recall of brief stories in the East Boston Memory Test, were standardized (using the mean and standard deviation from all subjects at baseline evaluation) and averaged to construct an Episodic memory domain.

#### The National Institute on Aging Late-Onset Alzheimer Disease Family Based Study (NIA-LOAD FBS)

The NIA-LOAD, a family-based study, is a collaboration among Alzheimer Disease Centers (ADC) in the United States with recruitment criteria that included families with multiple members affected by LOAD [38].

Assessment of Cognitive Function. The episodic memory domain scores were computed as the average of two standardized individual cognitive tests, immediate and delayed recall of Story A from the Wechsler Memory Scale Revised, as described previously [39]. To avoid data correlations due to the family-based nature of the NIA-LOAD cohort, we randomly selected a single individual from each family.

#### The National Alzheimer's Coordinating Center (NACC)

The NACC, a longitudinal cohort study of Alzheimer's disease, was established by the National Institute on Aging in 1999 to facilitate collaborative research by using data collected from the approximately 30 NIA-funded Alzheimer’s Disease Centers (ADCs) across the United States [40]. Research use of the NACC database was approved by the University of Washington’s Institutional Review Board.

Assessment of Cognitive Function. The episodic memory domain is measured by two different tests from the Uniform Data Set (UDS) neuropsychological battery: Logical Memory Story A Immediate, and Delayed recall. Individual memory scores were standardized (using the mean and standard deviation from non-cognitively impaired subjects at baseline evaluation) and then averaged to obtain the Episodic memory domain.

#### The Religious Orders Study and Rush Memory and Aging Project Alzheimer’s Disease Center cohorts (ROSMAP)

Study participants were drawn from two different population-based cohorts: i) The Religious Orders Study (ROS) study, which includes older Catholic nuns, priests, and brothers from groups across the United States and ii) The Rush Memory and Aging Project (MAP), which includes older individuals from the metropolitan Chicago area. At the time of enrollment, participants were at least 50 years old and non-demented. Detailed description of the cohorts can be found elsewhere [41, 42]. The study was approved by the Institutional Review Board of Rush University Medical Center and all participants signed an informed consent. Both cohorts share a large common core of data with evaluations conducted by the same study team allow efficient merging of items and tests for analyses.

Assessment of Cognitive Function. As previously described [43], an episodic memory domain consisted of seven measures of memory: immediate and delayed recall of story A from Logical Memory and of the East Boston Story and Word List Memory, Word List Recall, and Word List Recognition. Individual cognitive tests were standardized (using the mean and standard deviation from the sample of non-demented subjects at baseline) and averaged to obtain the episodic memory domain.

### Statistical analysis

#### Quality control data management

Criteria to be included in the current analyses were as follows: i) number of follow-up evaluations ranging from a minimum of two to a maximum of 15, ii) have available data on episodic memory scores, sex, age, and education, and iii) have available Genome-Wide Association Study (GWAS) data for future genetic analysis. The quality control exclusion criteria for study participants included: duplicated follow-up evaluations within the same year, dementia diagnosis at a specific visit which subsequently reverted to non-cognitive impairment, missing values for education and, younger than age 65.

#### Baseline samples for analysis

Primary and secondary statistical analyses were conducted using two different samples defined based on dementia status at baseline evaluation: i) no-cognitive impairment sample (NCI), which includes individuals without cognitive impairment at baseline evaluation and ii) all individuals sample (AI) which includes all study participants at baseline evaluation regardless of their dementia status. The differences in socio-demographic characteristics of the study participants (sex, age, education and ethnic background) between NCI and AI samples were assessed using likelihood ratio chi-square tests for the dichotomous variables and ANOVA tests for the continuous variables).

#### Computation of years from baseline and total years of follow up

We computed two additional variables, years from baseline and total years of follow-up. For each study participant, the years from baseline variable at a specific visit was computed as the number of years passed from the participant’s first evaluation (baseline) to the visit being considered. The total years of follow-up variable was computed as the total number of years that the study participant was followed-up.

#### Regression based models for EM scores adjustments

Socio-demographic variables can have an impact on the scores on a variety of neuropsychological measures including memory [44]. We used linear mixed models to adjust EM domain scores for sex, age, and education. Additional adjustment included the EM scores at baseline evaluation (EM_BA_) and study site when all cohorts were analyzed together. The linear regression residuals of the episodic memory scores (EM_res_) were then used as outcome in the LCMM models and downstream analyses.

#### Primary analyses: The Latent Class Mixed Model (LCMM)

The Latent Class Mixed Model [45] was used to assess the latent profiles of episodic memory trajectories. LCMM uses a mixed effects model with fixed and random effects terms to capture the characteristics of EM performance over-time [46]. The fixed effect term considers all individuals from the entire study sample to estimate the EM parameters, including mean slope and mean intercept, which characterize the differences in over-time EM performance between individuals. On the other hand, the random effect term estimates the variance of the EM parameters, the intercepts and slopes around the fixed effect term for each study participant to model the differences in over-time EM performance within individuals. LCMM fixed and random effects terms included total years of follow-up and years from baseline respectively as predictors of the latent class structure. LCMM estimation was performed using a maximum likelihood method and the optimal number of latent classes was empirically determined based on Bayesian information criterion.

When more than two clusters (EMT_Stables_/EMT_Decliners_) were estimated, we reran LCCM analyses fixing the number of latent class to two for an easier interpretation of the results.

The LCMM algorithm allows to model up to four different continuous link functions to relate the observed outcome and the underlying latent process [47]. We specified the standard linear mixed model. This link function yields parameter estimates in a different scale of the episodic memory scores (with mean equal to 0 and variance equal to 1). To obtain parameter estimates, we conducted a post-hoc analysis of the EMTs clusters estimated by LCMM (EMT_Stables_/EMT_Decliners_) using linear mix models.

1. Primary analyses: LCMM within study cohorts. The primary LCMM analysis did not consider an integrative data approach (i.e., pooling data from all study cohorts) based on the substantial methodological differences between cohorts (sampling frameworks, design characteristics, socio-ethnic-demographic characteristics, etc.). These multiple sources of between-studies heterogeneity may have a strong impact in the viability of the inferences we draw from pooled datasets [48, 49]. Therefore, EMTs estimation via LCMM algorithm was run independently within each of the study cohorts.
2. Primary analyses: LCMM across study cohorts stratified by sex and ethnic background. An integrative data approach was used to assess the impact of sex, ethnic background and education in the EMTs. Pooled data LCMM analyses were performed within five different stratum: sex (women, men), and ethnicity, which included three groups: non-Hispanic whites (NHW), African-Americans (AfAm) and Caribbean-Hispanics (CH).

As previously described, linear regression residuals of EM scores (EM_res_) were used as outcome in the LCMM. In the sex stratified LCMM analysis, the EM scores were residualized based on age, education, EM_BA_, YB and TY. In the ethnicity stratified LCMM analysis, residualization of EM raw scores included age, sex, education, EM_BA_, YB and TY.

#### Secondary analyses: Predictors of episodic memory progression over-time

A linear mixed models (LMM) framework was used to evaluate the impact of socio-economic (sex, education and ethnicity) and a genetic factor (*APOE* genotype) in the EMTs. The linear mixed models used the slope of the residualized episodic memory scores (EM_res_) as a continuous outcome and the socio-economic and *APOE* gene as independent variables. The models incorporated an interaction terms between the independent factor tested and years from baseline for which statistical significance is reported. The effect of education on EMT was analyzed within each ethnic group, due to differences in educational attainment across ethnic groups. The years of education were dichotomized into two different categories: i) lower education level for those study participants with less than 14 years of education and ii) high education level for those study participants with 14 years of education or more. For the purpose of these analyses, NHW ethnicity was defined using all Non-Hispanic White cohorts except for NIA-LOAD and NACC cohorts. By using only population-based cohorts (WHICAP, CHAP, ROSMAP), we tried to minimize the potential sampling bias associated with the different recruitment of the two LOAD cohorts.

Secondary analyses: Age-stratified Incident Rates of Dementia. Incidence rates of dementia were computed in the sample that included only individuals without cognitive impairment. Incidence rates were calculated by dividing the number of new cases with onset of dementia by the number of person-years at risk in each age group considered [50]. Person-years were calculated from the time of study entry for each individual until the time of dementia or until the date of the last examination for those who remained unaffected (including dates of death, loss to or unavailability for follow-up, or the most recent contact). Incidence rates were stratified within three age categories (65–74, 75–84, and ≥85 years). Incidence rates were reported as number of dementia cases per year and per 100 people. Confidence intervals (95%) for the incidence rate were computed assuming a Poisson distribution for the number of new cases in each age group. We additionally conducted a sensitivity analysis, in which the dementia incident rates were re-computed after excluding non-population based study cohorts (NACC and NIA-LOAD).

## Results and discussion

Study cohorts. Characteristics of each study cohort before exclusion criteria was applied are summarized in S1 Table. After these exclusions the final sample size was 13,041 persons for the all individuals at baseline, and 10,221 persons for the non-cognitive impaired individuals at baseline. Table 1 summarizes characteristics of the study participants at NCI and AI baseline samples across all study cohorts.

**Table 1:** Characteristics of the demented and non-demented study participants at baseline evaluation across all study cohorts.

	demented	NCI	
Variables	n = 2815	n = 10,222	p
women (%)	1,339 (20)	5,237 (80)	<0.001
age (average± SD)	78 ± 7	74 ± 7	<0.001
education (average± SD)	15 ± 4	14 ± 4	<0.001
Non-Hispanic White (%)	2,564 (27)	7,049 (73)	<0.001
African-American (%)	117 (5)	2,477 (95)	<0.001
Caribbean-Hispanic (%)	134 (16)	696	<0.001
*APOE*-ε4 (%)	1,471 (37)	2,495 (63)	<0.001

Cohort-specific definitions of the EM domain along with the average and standard deviation values of EM scores are provided in S2 Table.

The characteristics of the participants within each of the study cohorts are summarized in Table 2.

**Table 2:** Study participant characteristics within each of the cohorts.

Study cohort	Baseline sample	N	% women	age (avg ± SD)		education	%*APOE* E4	ETMs	
								EMT_Stables_	EMTs_Decliners_
				BA	LE	(avg ± SD)		N (%)	N (%)
WHICAP_AfAm	NCI	558	73	75 ± 6	82± 6	13 ± 3	31	454 (81)	104 (17)
	AI	624	72	76± 6	82 ± 6	12 ± 3	30	583 (93)	41 (7)
WHICAP_CH	NCI	696	70	75 ± 6	82± 6	8 ± 4	20	358 (51)	338 (49)
	AI	830	69	75 ± 6	82 ± 6	8 ± 4	22	783 (94)	47 (6)
WHICAP_NHW	NCI	571	61	76 ± 6	83 ± 6	14 ± 3	18	560 (98)	11 (2)
	AI	589	60	76 ± 6	83 ± 6	14 ± 3	18	579 (98)	10 (2)
CHAP_NHW	NCI	1,275	62	72 ± 6	80 ± 7	15 ± 3	13	1,244 (98)	31 (2)
	AI	1,298	62	73 ± 6	81 ± 7	15 ± 3	13	1,246 (96)	52 (4)
CHAP_AfAm	NCI	1,919	63	73 ± 4	77 ± 5	12 ± 3	32	1,063 (55)	856 (45)
	AI	1,970	63	73 ± 4	77 ± 5	12± 3	32	1,861 (94)	109 (6)
NACC-ADGC	NCI	3,276	64	77 ±8	79 ± 8	16 ± 6	29	3,068 (94)	208 (6)
	AI	5,355	58	77± 7	79 ± 8	16 ± 5	34	468 (9)	4,887 (91)
NIA-LOAD	NCI	640	62	77 ± 7	79 ± 7	15 ± 3	31	350 (55)	290 (45)
	AI	691	60	77 ± 7	79 ± 7	15 ± 3	34	327 (47)	364 (53)
ROSMAP_NHW	NCI	1,285	75	78 ± 7	84 ± 7	17 ± 4	17	1,058 (82)	227 (18)
	AI	1,680	72	80 ± 7	85 ± 7	16 ± 4	21	945 (56)	735 (44)

AfAm: African-American; CH: Caribbean-Hispanic; NHW: Non-Hispanic White; BA: baseline evaluation; LE = last evaluation; EMT: Episodic Memory Trajectory

The average age of the participants across all cohorts was 75±6 at baseline evaluation and 81±7 at the last evaluation. The average years of education varied depending on the participant’s ethnic background: Non-Hispanic Whites (15±1), African-Americans (13±1) and Caribbean-Hispanics (8±4). The percentage of women ranged from 58% to 72% in the IA sample and from 61% to 75% in the NCI sample. The frequency of the *APOE*-ε4 allele was very similar in both NCI and AI samples, ranging from 13% to 32% and from 13% to 34%, respectively.

As shown in Table 2, within each of the study cohorts, the majority of the study participants in the NCI baseline sample clustered into the EMT_Stables_ cluster, ranging from 51% to 98%. Similar patterns were observed in the AI baseline sample, except for NACC cohort, where majority of participants were aggregated into EMT_Decliners_ cluster.

**Fig 1:**
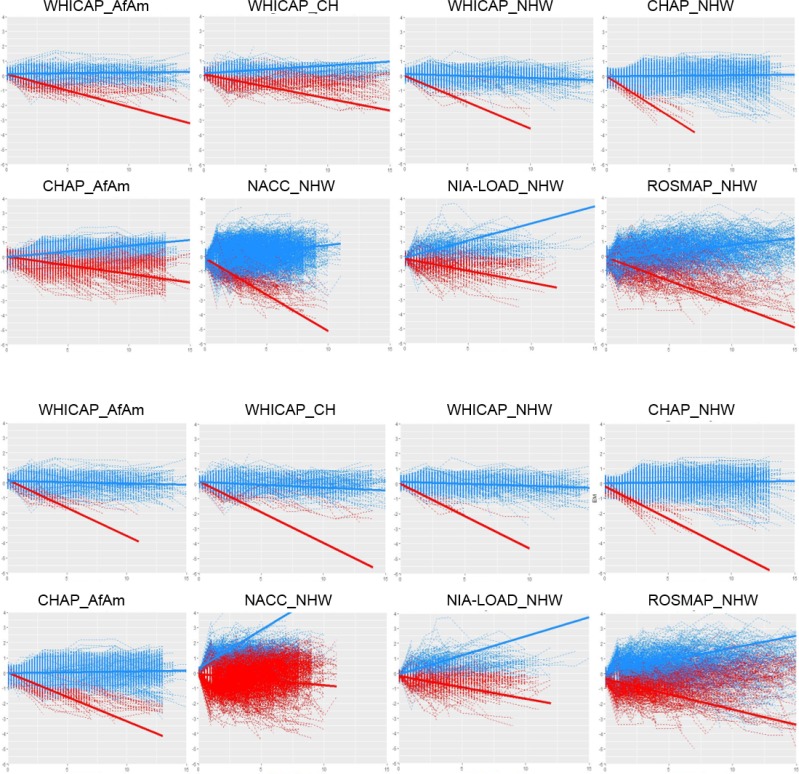
**Episodic memory trajectories considering non-cognitively impaired subjects (two upper panels) and all subjects (two lower panels) at baseline within each of the study cohorts.** NHW: Non-Hispanic Whites; AfAm: African-Americans; CH: Caribbean-Hispanics. The X-axis correspond to the time of follow-up in years (ranging from 0 to 15); the Y-axis correspond to the residual episodic memory score (ranging from -6 to 4) after being adjusted for sex, age, education, episodic memory scores at baseline and total years of follow-up (truncated to a maximum of 15 years).

When primary LCMM analyses were restricted to NCI baseline sample (Fig 1), two EMTs clusters were estimated within each of the study cohorts: individuals who exhibited either a constant memory function or whose memory function improved (EMT_Stables_), and individuals who exhibited memory decline (EMT_Decliners_). The same qualitative clustering solution (EMT_Stables_ and EMT_Decliners_) was observed when analyses were repeated in the AI baseline sample (Fig 1). To evaluate the extent of overlap between the two baseline samples (AI versus NCI), we tabulated the number (%) of participants in EMT_Stables_ and EMT_Decliners_ clusters (S3 Table). Overall study cohorts, the majority of the study participants classified as EMT_Stables_ within the NCI baseline sample analysis remain classified as Stables in the AI baseline sample (63%), and majority of EMT_Decliners_ also remain classified as Decliners when the dementia cases are included (58%).

Post-hoc linear mixed models within each of the study cohorts (S4 Table) demonstrated that, in both NCI and AI baseline samples, the average estimate of the EM_res_ slope in the EMT_Decliners_ clusters declined significantly over time (p<0.05) when compared of the decay in average EM_res_ slope in the EMT_Stables_ cluster We also observed significant heterogeneity in the estimates of the EM_res_ intercepts.

We also evaluated whether EMTs clusters derived from LCMM analyses of the pooled datasets might differ by sex (S1 Fig) or ethnic background (S2 Fig) by stratifying the sample into two sex groups (women and men) and into three different ethnic groups (Non-Hispanic Whites, African-Americans and Caribbean-Hispanics).

Post-hoc parameter estimation within stratum (S5 Table) showed differences in the average EM_res_ slope when EMT_Stables_ and EMT_Decliners_ clusters were compared across ethnic groups and sex. In both NCI and AI baseline samples, average EM_res_ slope of women appeared to be steeper than men, and the steeper decay in the average slope of EM_res_ across ethnicities is observed in AfAm.

Secondary analyses testing whether the observed differences in the primary analyses were statistically significant (Table 3) did not show significant interaction between sex and the per-year decay of EM_res_ slope in neither EMT cluster. We did find a statistically significant interaction between per-year decay of EM_res_ slope and ethnicity, education, and *APOE* genotype. Within the EMT_Stables_ cluster, the decay of EM_res_ slope appeared to be steeper in AfAm compared to NHW (p = 4.7 x 10^−7^) and in CH compared to NHW (p = 4.3 x 10^−15^). When comparing AfAm and CH, decay of EM_res_ slope appeared to be steeper in CH in the EMT_Stables_ cluster (p = 4.0 x10^-10^). The lack of significance in the above interactions within the EMT_Decliners_ cluster is most likely due to the reduced statistical power because of limited sample size of the EMT_Decliners_ clusters across the three different comparison groups.

**Table 3:** Secondary analyses in the non-cognitive impaired (NCI) baseline sample.

Strata	strata_groups	N		total	P_interaction_	
		EMT_Stables_	EMT_Decliners_		EMT_Stables_	EMT_Decliners_
sex	man	4,542	442	4,984	0.331	0.964
	women	4,781	455	5,236		
	total	9,323	897	10,220		
ethnicity	NHW	2,943	188	3,131	***4*.*7E-07***	0.050
	AfAm	2,430	47	2,477	*** ***	
	total	5,373	235	5,608	*** ***	
	NHW	2,967	164	3,131	***4*.*3E-15***	0.550
	CH	693	3	696	*** ***	
	total	3,660	167	3,827	*** ***	
	AfAm	1,549	928	2,477	***4*.*0E-10***	0.626
	CH	399	297	696		
	total	1,948	1,225	3,173		
education_NHW	low education	1,073	51	1,124	***1*.*6E-07***	0.051
	high education	1,895	112	2,007		
	total	2,968	163	3,131		
education_AfAm	low education	1,550	113	1,663	0.085	0.150
	high education	777	37	814		
	total	2,327	150	2,477		
education_CH	low education	313	306	619	0.457	0.467
	high education	45	32	77		
	total	358	338	696		
*APOE*	non-carriers	1,890	1,998	3,888	***4*.*2E-07***	0.222
	E4 carriers	640	770	1,410		
	total	2,530	2,768	5,298		

There was also a significant interaction between decay of EM_res_ slope and education in the EMT_Stables_ cluster. Among NHW, decay of EM_res_ slope appeared to be steeper in study participants with low education levels compared to those with higher educational attainment (p = 1.6 x 10^−7^). We did not observe a statistically significant effect of education in EMT_Stables_ in AfAm and CH.

Finally, we observed a strong effect of the *APOE* genotype in both EMT clusters. The decay of EM_res_ per year was steeper in non-carriers of *APOE*-E4 allele compared to carriers. (p = 4.4 x 10^-07^and 2.4 x 10^−07^, for EMT_Stables_ and EMT_Decliners_ respectively).

Not unexpectedly, the highest incidence rates of dementia were observed in the oldest age group (≥85 years old, Table 4).

**Table 4:** Age-specific annual incident rates of incident dementia.

strata	var	age group	n	ADcases	Total_PY_	IR	95% CI
Sex	Women	65–74	1,273	99	4,909	0.02	0.01–0.02
		75–84	2,492	397	15,140	0.03	0.02–0.03
		≥85	1,470	417	10,650	0.04	0.03–0.05
			5,235				
	Men	65–74	1,175	86	4,939	0.02	0.01–0.03
		75–84	2,368	352	15,147	0.02	0.02–0.03
		≥85	1,442	415	10,623	0.04	0.03–0.05
			4,985				
Ethnicity	NHW	65–74	1,532	97	5,615	0.02	0.02–0.03
		75–84	3,167	426	17,839	0.02	0.02–0.03
		≥85	2,348	619	15,852	0.04	0.03–0.05
			7,047				
	AfAm	65–74	767	54	3,810	0.01	0.01–0.02
		75–84	1,357	195	10,668	0.02	0.01–0.03
		≥85	353	110	3,537	0.03	0.01–0.05
			2,477				
	CH	65–74	149	34	423	0.08	0.04–0.13
		75–84	336	128	1,780	0.07	0.04–0.10
		≥85	211	103	1,884	0.05	0.02–0.09
			696				
EMTs	Stable	65–74	1,885	66	7,427	0.01	0.01–0.01
		75–84	3,855	339	24,016	0.01	0.01–0.01
		≥85	2,414	480	18,157	0.03	0.02–0.04
			8,154				
	Decliners	65–74	563	119	2,421	0.05	0.02–0.09
		75–84	1,005	410	6,271	0.07	0.04–0.10
		≥85	498	352	3,116	0.11	0.09–0.15
			2,066				

ADcases: subjects diagnosed with Alzheimer’s disease; Total_PY_: total number of persons-years to either dementia or non-dementia; IR: incident rate of dementia per year and per 100 people; CI: Confidence intervals.

Within this age stratum, the highest incidence rates of dementia were observed among the subjects with Caribbean-Hispanics ancestry (5% per year). Stratifying by EMTs clusters, those with a declining episodic memory trajectory were four times more likely to develop dementia compared with those whose episodic memory scores remained stable (11% per year versus 3% per year). Sensitivity analyses results using only population-based cohorts revealed an even more pronounced difference, EMT_Decliners_ were almost six times more likely to develop dementia compared with EMT_Stables_ (data not shown)_._

Analysis of episodic memory trajectories in a large sample of ethnically diverse older adults identified two major clusters: EMT_Stables_, consisting of individuals whose memory function remains stable or improved over time, and EMT_Decliners_, consisting of individuals whose memory function declined. Compared to those with Stable trajectory, individuals characterized as Decliners exhibited a significant decline of episodic memory performance over time, were more likely to have non-white ethnic background, fewer years of education, a higher frequency of ε4 allele at *APOE* gene and five times more likely to develop dementia.

Consistent with previous studies, the majority of the cognitively healthy participants at baseline evaluation (from a minimum of 51% to a maximum of 98%) maintain their memory performance over time [3, 10, 11]. A similar pattern was observed when individuals with dementia were included in the baseline evaluation, i.e., the majority of the study participants clustered into the EMT_Stables_ trajectory (from a minimum of 47% to a maximum of 98%). The exception to this pattern were NACC and NIA-LOAD cohorts, in which the majority of subjects clustered into the EMT_Decliners_ trajectory. Unlike the other population-based study cohorts, participants from NACC and NIA-LOAD cohorts are enrolled based on late onset Alzheimer’s disease diagnosis or potential increased risk of developing AD dementia, therefore, we expect a higher proportion of Decliners.

Our results did not find a statistically significant interaction between sex and the decay of episodic memory over time. The literature regarding to the relationship between sex and decline of cognitive function has been inconsistent. Some longitudinal studies reported no sex differences and parallel patterns of decline [51], while others have argued that women are either especially vulnerable to memory decline [52] or exhibited greater resilience to age-related cognitive decline [12, 53]. Methodological differences such as sampling bias (under-sampling men or women) or analytical approaches may help to explain the contradictory findings. We also found slightly higher incidence rates of dementia among women in the oldest age group, although this difference did not achieve statistical significance.

Conversely, we did observe a strong interaction effect between decay of episodic memory and ethnicity, education, and *APOE* genotype. The decay of EM_res_ slope appear to be steeper in non-white ethnic groups compared to non-Hispanic whites, with a steeper decline among Caribbean-Hispanics (p = 4 x10^-10^). As reported in the WHICAP cohort [33], a Caribbean-Hispanic population appears to have a higher burden of dementia. Ethnic differences in incident rates of dementia have been attributed to differences in biological risk factors (i.e., cerebrovascular disease), differential exposure to environmental risk factors, or genetic risk among other factors. Future work may benefit from using genetic data to define ancestry, in addition to self-reported ethnic classifications [54].

Our results showed that those with lower education had higher odds of being Decliners. The strongest effect of education was observed among non-Hispanic whites clustered as EMT_Stables_. The decay of EM_res_ slope was steeper in those study participants with low education levels (p = 1.6 x 10^−7^). Findings with respect to whether educational attainment moderates the trajectory of age-related cognitive decline have been mixed. Studies in older adults without dementia reported that educational attainment attenuates cognitive decline [55]. These results support the hypothesis of cognitive reserve [56], which suggest that educational attainment may supply a set of skills that allows to tolerate the age-related changes and disease-related pathology in the brain without developing clinical symptoms or signs of disease. However, other reports [57] have found that cognitive decline in individuals with greater educational attainment occurs at a similar rate as in individuals with less education. Interestingly, other studies that allow for a random change point in the rate of decline reported that education was associated with a slower rate of decline, a delayed change point, followed by a faster rate of decline [58].

Finally, we observed a strong effect of the *APOE* genotype. Study participants who carry one or two copies of the ε4 allele at the *APOE* gene displayed a steeper decline of episodic memory than those who do not carry any ε4 allele. APOE_ε4 has been consistently shown in previous studies to be related to cognitive decline, particularly episodic memory. Results from a meta-analysis [59] of 40,942 cognitively healthy adults showed that carriers of *APOE*_ε4 perform significantly worse on measures of episodic memory, and that the differences between carriers and non- ε4 carriers become larger with increasing age.

Study limitations. Our study has some limitations. First, our analyses were not adjusted for practice effect, i.e., improvement of memory performance because of repeated exposure to cognitive assessments. Since the learning effect is confounder of aging, there is probably a learning effect component in both EMT groups, i.e., the EMT_Stables_ includes both learning and aging effects, while the difference between Stable and Decliners groups includes the pathological related cognitive performance beyond the learning and normal aging effect. Moreover, there is no empirical evidence that practice may result in different estimated associations between exposure to cognitive tests and rate of cognitive change [60]. Second, the individual neuropsychological tests used to assess episodic memory performance varied from cohort to cohort. Nonetheless, the factors that modulate the episodic memory trajectories were consistent across the study cohorts, suggesting that the findings are reliable. Third, the diagnosis of cognitive status across of the study cohorts did not differentiate non-cognitively impaired as either normal or mild cognitive impaired, therefore, it is possible that study participants have been misclassified. Fourth, additional factors not assessed in the study such as cardiovascular, metabolic or mental health, as well as other types of life-style factors, could also contribute to cognitive decline.

The study also had several strengths. To our knowledge, the present study represents the largest (n = 13,037) cohort for which trajectories of memory performance over time have been derived. Moreover, the study includes a sample of two minority populations underrepresented in research studies, African-Americans and Caribbean-Hispanics.

## Conclusions

Analysis of episodic memory performance over time in a large sample of ethnically diverse older adults, allowed us to cluster study’s participants into distinct episodic memory trajectories. Different socio-economic factors including age and education, along with *APOE* genotype, and dementia risk modulate these episodic memory trajectories. Additional research is needed to further elucidate additional risk and/or resilience factors within these trajectories.

Future research should focus on identifying risk and protective factors that contribute to this differential rate of incident dementia across the episodic memory trajectories. Special emphasis should be place on evaluating the extent to which such risk or resilience results from genetic predisposition.

## Supporting information

S1 Fig Episodic memory trajectories (EMTs) stratified by sex across all study cohorts. **A) only non-cognitively impaired subjects at baseline; B) all subjects at baseline**. The X-axis correspond to the time of follow-up in years (ranging from 0 to 15); the Y-axis correspond to the residual episodic memory score (ranging from -6 to 4) after being adjusted for sex, age, education, episodic memory scores at baseline and total years of follow-up (truncated to a maximum of 15 years).
(EPS)

S2 Fig Episodic memory trajectories (EMTs) stratified by ethnicity. **A) only non-cognitively impaired subjects at baseline; B) all subjects at baseline**. The X-axis correspond to the time of follow-up in years (ranging from 0 to 15); the Y-axis correspond to the residual episodic memory score (ranging from -6 to 4) after being adjusted for sex, age, education, episodic memory scores at baseline and total years of follow-up (truncated to a maximum of 15 years).
(EPS)

S1 Table Characteristics of the original study cohorts before exclusion criteria. (DOCX)

S2 Table Definition of episodic memory domain across study cohorts. BA corresponds to the episodic memory scores at baseline evaluation; LE corresponds to episodic memory scores at last evaluation.
(DOCX)

S3 Table Episodic memory trajectories (EMTs) distribution under the two analyses models (NCI and AI baseline samples). (DOCX)

S4 Table Parameters estimates from post-hoc linear mixed models using the trajectories from LCMM for all subjects at baseline evaluation. (DOCX)

S5 Table Parameters estimates from post-hoc linear mixed models using the trajectories from LCMM stratified by sex and ethnicity for all subjects at baseline. (DOCX)
