# Supplementary material for: Episodic memory performance in a multi-ethnic longitudinal study of 13,037 elderly
Source: PLoS One. 2018 Nov 21;13(11):e0206803. doi: 10.1371/journal.pone.0206803 (PMC6248922; doi:10.1371/journal.pone.0206803)
Supplement: S1 Table — (DOCX) [file pone.0206803.s003.docx]

|  |  |  |  | age (avg ± SD) | | education | YB | | number of visits | | single visit |
| --- | --- | --- | --- | --- | --- | --- | --- | --- | --- | --- | --- |
| Study cohort | Ethnicity | N | % women | BA | LE | (avg ± SD) | min-max | (avg ± SD) | min-max | avg ± SD | N (%) |
| WHICAP | AfAm | 2,208 | 71 | 77±7 | 80±8 | 11±4 | 0-26 | 3±4 | 1-14 | 3±2 | 758 (34) |
|  | CH | 3,248 | 69 | 76±7 | 80±8 | 7±5 | 0-25 | 4±5 | 1-14 | 3±2 | 972 (30) |
|  | NHW | 1,866 | 62 | 77±8 | 80±9 | 13±4 | 0-22 | 4±4 | 1-12 | 3±2 | 592 (32) |
| CHAP | NHW | 1,335 | 62 | 73±6 | 80±7 | 14±3 | 0-16 | 6±3 | 1-5 | 3±1 | 9 (1) |
|  | AfAm | 2,324 | 62 | 70±4 | 77±6 | 12±3 | 0-16 | 6±3 | 1-5 | 3±1 | 181 (8) |
| NACC | NHW | 11,057 | 60 | 75±9 | 79±9 | 16±7 | 0-13 | 5±3 | 1-12 | 4±3 | 1836 (17) |
| NIA-LOAD | NHW | 2,945 | 62 | 74±8 | 77±9 | 14±3 | 0-30 | 6±6 | 1-13 | 3±1 | 163 (6) |
| RADC | NHW | 3,724 | 64 | 78±8 | 85±8 | 16±4 | 0-23 | 7±6 | 1-24 | 8±5 | 327 (9) |
