# Supplementary material for: Episodic memory performance in a multi-ethnic longitudinal study of 13,037 elderly
Source: PLoS One. 2018 Nov 21;13(11):e0206803. doi: 10.1371/journal.pone.0206803 (PMC6248922; doi:10.1371/journal.pone.0206803)
Supplement: S2 Table — BA corresponds to the episodic memory scores at baseline evaluation; LE corresponds to episodic memory scores at last evaluation. (DOCX) [file pone.0206803.s004.docx]

|  |  |  | NCI | | AI | |
| --- | --- | --- | --- | --- | --- | --- |
| Cohort | Ethnicity | cognitive tests | avg_BA_ ± SD | avg_LE_ ± SD | avg_BA_ ± SD | avg_LE_ ± SD |
| WHICAP | AfAm | average of the standardized tests | 0.50 ± 0.60 | 0.05 ± 0.85 | 0.35 ± 0.72 | -0.10 ± 0.93 |
|  | CH | (STR total immediate recall, delayed recall, and | 0.38 ± 0.48 | -0.05 ± 0.76 | 0.21 ± 0.61 | -0.20 ± 0.82 |
|  | NHW | delayed recognition) | 0.70 ± 0.60 | 0.23 ± 0.85 | 0.65 ± 0.66 | 0.19 ± 0.89 |
|  |  |  |  |  |  |  |
| CHAP | NHW | average of the standardized tests (LogMem IA,IIA) | 0.68 ± 0.58 | 0.55 ± 0.85 | 0.64 ± 0.65 | 0.50 ± 0.91 |
|  | AfAm |  | 0.50 ± 0.60 | 0.33 ± 0.85 | 0.45 ± 0.68 | 0.28 ± 0.90 |
|  |  |  |  |  |  |  |
| NACC | NHW | average of the standardized tests (LogMem IA,IIA) | 0.07 ± 1.00 | 0.16 ± 1.20 | -0.94 ± 1.61 | -0.94 ± 1.80 |
|  |  |  |  |  |  |  |
| NIA-LOAD | NHW | average of the standardized tests (LogMem IA, IIA) | -0.01 ± 0.96 | 0.09 ± 1.19 | -0.16 ± 0.11 | -0.09 ± 1.34 |
|  |  |  |  |  |  |  |
| RADC | NHW | average of the standardized tests (LogMem IA, IIA) | 0.05 ±0.99 | -0.06 ± 1.47 | -0.33 ± 1.68 | -0.55 ± 1.68 |
