# Supplementary material for: Episodic memory performance in a multi-ethnic longitudinal study of 13,037 elderly
Source: PLoS One. 2018 Nov 21;13(11):e0206803. doi: 10.1371/journal.pone.0206803 (PMC6248922; doi:10.1371/journal.pone.0206803)
Supplement: S3 Table — (DOCX) [file pone.0206803.s005.docx]

|  | AI baseline sample | |  |  |  |
| --- | --- | --- | --- | --- | --- |
|  | EMT_Stables_ | | EMTs_Decliners_ | |  |
| NCI baseline sample | n | % | n | % | total |
| EMT_Stables_ | 5,173 | 63 | 2,982 |  | 8,155 |
| EMT_Decliners_ | 869 |  | 1,196 | 58 | 2,065 |
| total | 6,042 |  | 4,178 |  | 10,220 |
