# Supplementary material for: Episodic memory performance in a multi-ethnic longitudinal study of 13,037 elderly
Source: PLoS One. 2018 Nov 21;13(11):e0206803. doi: 10.1371/journal.pone.0206803 (PMC6248922; doi:10.1371/journal.pone.0206803)
Supplement: S4 Table — (DOCX) [file pone.0206803.s006.docx]

|  |  | EMT_Stables_ |  | EMTs_Decliners_ | | Comparison | |
| --- | --- | --- | --- | --- | --- | --- | --- |
| Cohort | parameters | Intercept | Slope | Intercept | Slope | Intercept | slope |
| WHICAP_AfAm | Estimate | 0.14 | -0.04 | 0.14 | -0.30 | 0.00 | -0.26 |
|  | SD | 0.02 | 0.01 | 0.05 | 0.02 | 0.03 | 0.01 |
|  | P | <0.001 | <0.001 | 0.01 | <0.001 | 0.01 | <0.001 |
| WHICAP_CH | Estimate | 0.11 | -0.03 | 0.02 | -0.29 | -0.09 | -0.26 |
|  | SD | 0.02 | 0.01 | 0.05 | 0.02 | 0.03 | 0.01 |
|  | P | <0.001 | <0.001 | 0.638 | <0.001 | 0.64 | <0.001 |
| WHICAP_NHW | Estimate | 0.12 | -0.05 | -0.08 | -0.37 | -0.21 | -0.32 |
|  | SD | 0.02 | 0.01 | 0.10 | 0.36 | 0.07 | 0.35 |
|  | P | <0.001 | <0.001 | 0.392 | <0.001 | 0.39 | <0.001 |
| CHAP_NHW | Estimate | 0.02 | 0.01 | -0.27 | -0.45 | -0.29 | -0.46 |
|  | SD | 0.02 | 0.01 | 0.06 | 0.02 | 0.03 | 0.01 |
|  | P | 0.421 | 0.023 | <0.001 | <0.001 | -0.42 | -0.02 |
| CHAP_AfAm | Estimate | 0.00 | 0.01 | -0.08 | -0.25 | -0.08 | -0.26 |
|  | SD | 0.02 | 0.01 | 0.04 | 0.01 | 0.02 | 0.00 |
|  | P | <0.001 | <0.001 | 0.067 | 0.000 | -0.02 | -0.01 |
| NACC_NHW | Estimate | 0.05 | 0.10 | -0.16 | -0.34 | -0.22 | -0.44 |
|  | SD | 0.02 | 0.00 | 0.03 | 0.00 | 0.01 | 0.00 |
|  | P | <0.001 | <0.001 | <0.001 | <0.001 | <0.001 | <0.001 |
| NIA-LOAD | Estimate | 0.13 | 0.45 | -0.19 | 0.03 | -0.33 | -0.41 |
|  | SD | 0.09 | 0.04 | 0.00 | 0.02 | -0.09 | -0.03 |
|  | P | 0.136 | <0.001 | <0.001 | <0.001 | -0.14 | <0.001 |
| RADC_NHW | Estimate | 0.12 | 0.02 | -0.56 | -0.10 | -0.68 | -0.13 |
|  | SD | 0.02 | 0.01 | 0.02 | 0.01 | 0.00 | 0.00 |
|  | P | <0.001 | <0.001 | <0.001 | <0.001 | <0.001 | -0.09 |
