# Supplementary material for: Episodic memory performance in a multi-ethnic longitudinal study of 13,037 elderly
Source: PLoS One. 2018 Nov 21;13(11):e0206803. doi: 10.1371/journal.pone.0206803 (PMC6248922; doi:10.1371/journal.pone.0206803)
Supplement: S5 Table — (DOCX) [file pone.0206803.s007.docx]

|  |  | EMT_Stables_ |  | EMTs_Decliners_ | | Comparison | |
| --- | --- | --- | --- | --- | --- | --- | --- |
| Cohort | parameters | Intercept | Slope | Intercept | Slope | Intercept | slope |
| women | Estimate | -0.18 | 0.07 | -0.37 | -0.35 | -0.19 | -0.43 |
|  | SD | 0.01 | 0.01 | 0.03 | 0.01 | 0.02 | 0.01 |
|  | P | <0.001 | <0.001 | <0.001 | <0.001 | <0.001 | <0.001 |
| men | Estimate | -0.02 | 0.07 | -0.49 | -0.05 | -0.48 | -0.12 |
|  | SD | 0.01 | 0.01 | 0.01 | 0.01 | 0.00 | 0.00 |
|  | P | 0.202 | <0.001 | <0.001 | <0.001 | -0.20 | <0.001 |
| NHW | Estimate | -0.16 | 0.08 | -0.44 | -0.09 | -0.28 | -0.16 |
|  | SD | 0.01 | 0.00 | 0.01 | 0.01 | 0.00 | 0.00 |
|  | P | <0.001 | <0.001 | <0.001 | <0.001 | 0.00 | <0.001 |
| AfAm | Estimate | 0.10 | 0.04 | 0.02 | -0.20 | -0.08 | -0.25 |
|  | SD | 0.02 | 0.01 | 0.02 | 0.01 | 0.00 | 0.01 |
|  | P | <0.001 | <0.001 | 0.190 | <0.001 | 0.19 | <0.001 |
| CH | Estimate | 0.23 | -0.02 | 0.01 | -0.19 | -0.22 | -0.17 |
|  | SD | 0.02 | 0.01 | 0.02 | 0.01 | 0.00 | 0.00 |
|  | P | <0.001 | <0.001 | 0.667 | <0.001 | 0.67 | -0.06 |
